# Assessing disordered eating behaviours and attitudes: Factor structure and measurement invariance of the Arabic version of the eating attitudes test (EAT-26) in Saudi Arabia

**DOI:** 10.1186/s40337-024-01137-2

**Published:** 2024-11-19

**Authors:** Mohsen M. Alyami, Saeed A. Al-Dossary

**Affiliations:** Department of Psychology, College of Education, University of Ha’il, Ha’il, Saudi Arabia

**Keywords:** Eating attitudes test, EAT-26, Eating disorder, Disordered eating behaviours and attitudes, Anorexia Nervosa, Arabic

## Abstract

**Background:**

The factorial structure of the Eating Attitudes Test (EAT-26) has been found to be inconsistent across studies and samples. This study aimed to resolve inconsistencies in the factorial structure of the Arabic version of the EAT-26 by identifying the best-fitting model and test its measurement invariance across sexes and BMI categories in a large non-clinical Saudi sample.

**Methods:**

1,734 Saudi adults (*M*_age_ 26.88 and SD 9.13), predominantly female, completed an online survey. Several existing models were tested (e.g., original 26-item three-factor model, second order 26-item three-factor model, 20-item four-factor model, and 16-item four-factor model) using confirmatory factor analysis (CFA). Fit indices including the CFI, SRMR, and RMSEA were used to identify the best-fitting model for Arabic version of the EAT-26. Multi-group confirmatory factor analysis (MGCFA) was used to test measurement invariance.

**Results:**

The original three-factor model and two other common models demonstrated poor fit (e.g., CFI = 0.727; SRMR = 0.0911; RMSEA = 0.085 [90% CI 0.082–0.087] for the original three-factor model). Instead, a 16-item, four-factor structure [(Self-Perceptions of Body Weight), (Dieting), (Awareness of Food Contents), and (Food Preoccupation)] showed acceptable fit ([CFI = 0.904; SRMR = 0.0554; RMSEA = 0.073 [90% CI 0.068- 0.077]). Internal consistency was good (α and ω = 0.88), and measurement invariance was supported across sex (male and female) and BMI categories (underweight, normal weight, overweight, and obese).

**Conclusions:**

These findings underscore the need for culturally relevant validation of the EAT-26 among Arabic-speaking populations, as the revised factorial structure diverged from previously established models. Future research should further examine this revised 16-item, four-factor structure in clinical settings.

**Supplementary Information:**

The online version contains supplementary material available at 10.1186/s40337-024-01137-2.

## Abstract (plain language summary)

Disordered eating behaviours and attitudes are unhealthy behaviours and attitudes toward food and weight, which may include restrictive dieting, purging, excessive exercise, abuse of laxatives, distorted body image, and body dissatisfaction, all of which have been linked to eating disorders. This study investigated the reliability and validity of the Arabic version of the Eating Attitudes Test (EAT-26) among Saudi adults. Contrary to the original factor structure, findings showed that the EAT-26 Arabic version has a four-factor structure with 16 items. This revised 16-items version showed good fit indices and reliability. Researchers and clinicians should avoid using the original EAT-26 and instead use this newly proposed 16-item version in this demographic.

**Supplementary Information:**

The online version contains supplementary material available at 10.1186/s40337-024-01137-2.

## Background

Obesity is a significant global health issue. Saudi Arabia has been facing a rising prevalence of obesity over the past few decades, and this trend is likely to continue [58]. According to available data, the obesity rate in Saudi Arabia is among the highest in the world, with 23.7% of adults (aged ≥ 15 years) and 7.3% of children (< 15 years) being obese. Both females and males show comparable rates of obesity [28]. The negative impact of obesity on health and well-being is recognised by various stakeholders, including individuals with obesity, caregivers, and healthcare professionals [14]. Urbanisation, sedentary lifestyles, unhealthy eating habits, and a lack of physical activity have been cited as contributing to the high rates of obesity [46].

Obesity is closely linked to disordered eating behaviours and attitudes (DEBAs) as well as eating disorders (EDs). DEBAs encompass a wide range of unhealthy behaviours and attitudes toward food and weight that may not meet the criteria for a formal ED diagnosis, including restrictive dieting, purging, excessive exercise, abuse of laxatives, distorted body image, and body dissatisfaction [29, 53]. Results of studies with Saudi samples showed high DEBAs, especially among adolescents [6, 8, 24]. DEBAs may lead to the development of EDs such as anorexia nervosa, bulimia nervosa, and binge-eating disorder. These disorders are prevalent globally, with a lifetime prevalence of 0.16%, 0.63% and 1.53% respectively [54]. The Saudi National Mental Health Survey (SNMHS), a nationally representative population household survey, reported the 12-month prevalence of any of the three EDs at 3.2% and a lifetime prevalence of 6.1% which is higher than reported rates worldwide [5].

Given the high prevalence of EDs and DEBAs, reliable and valid screening tools becomes of high importance for research and clinical use. One commonly used screening tool to assess symptoms associated with DEBAs is the Eating Attitude Test (EAT). The EAT is a self-report measure that has two versions: the original EAT-40 [26] and its abbreviated form EAT-26 [27]. While the EAT was developed with clinical samples of adolescent females with AN, its use has since been expanded to diverse cross-cultural and non-clinical populations.

The EAT-26 consists of three factors: Dieting (i.e., 13 items related to avoidance of fatty foods and preoccupation with thinness), Bulimia and food preoccupation (i.e., 6 items related to thoughts about food and bulimia), and Oral control (i.e., 7 items related to self-control over food and societal pressure to gain weight) [27]. The EAT-26 has been used in a range of populations including adults with EDs and non-clinical samples [25], and has been translated into many languages including Chinese [34], French [39], Italian [22], Japanese [50], Russian [47], Spanish [55], and Urdu [33].

Despite the widely acknowledged reliability of the EAT-26, its factorial structure has been found to be inconsistent across studies and populations [56]. Although some studies replicated the same three-factor structure identified for the original English EAT-26 version [22], more recent research using different statistical methodologies has shown different factor structures of the EAT-26. A study with two independent samples of female college students compared the three-factor structure with 26 items [27] and a four-factor structure with 20 items [37]. The results showed an unacceptable model fit for the three-factor EAT-26 and a poor fit for the four-factor EAT-20. Four items that presented low factor loadings were eliminated and the four-factor model with 16 items was found to have an acceptable fit [51].

In a study conducted with a Russian non-clinical university female sample, the authors identified a five-factor model with 15 items that best fit the data [47]. A six-factor model with 18 EAT items was found to be a reliable and valid measure of DEBAs for an Irish adolescent sample [43]. Other research also found a six-factor model with 18 EAT items has the best fit among a large sample of French-speaking, ethnically diverse European and African participants using exploratory structural equation modelling (ESEM) [41]. Research using Rasch analysis with an adult sample of university students and adults undertaking a behavioural weight loss program found that a 19-item EAT version demonstrated a satisfactory fit in accordance with the expectations of the Rasch model [52].

Similarly, although the EAT-26 has been widely used among Arabic-speaking populations [1, 2, 7, 10, 23, 24], fewer studies have reported on its psychometric properties. For example, Al-Subaie et al. [9] validated the EAT-26 among Saudi young female students (grades 7–12) against diagnostic clinical interviews. The authors found that the Arabic EAT-26 exhibited a high false positive value (Al‐Subaie et al., [9]). No more information (e.g., internal reliability or factor structure) was reported by the authors.

The factorial structure of the Arabic EAT-26 version is unstable and inconsistent, similar to the criticisms levelled against the English and other language versions of the EAT-26. Mousa and Beretvas [49] examined the factor structure of the EAT-26 in a sample of adolescent schoolgirls in Jordan using exploratory factor analysis (EFA). The authors excluded three items due to low factor loadings and cross-loading, resulting in a 23-item with three factors [49], similar to the original structure model reported by Garner et al. [27]. A study using a large probability sample of predominantly young Qatari female university students identified a five-factor structure with 19 EAT items [36]. In a Lebanese community sample; however, a six-factor model had the best fit [30]. In this study, higher scores on the EAT-26 were associated with higher depressive symptoms, emotional eating, and starvation to reduce weight [30].

Overall, the EAT-26 appears to have different factors in different ethnic and cultural groups, most of which did not correspond with the original EAT-26 three-factor structure in terms of the number of factors and the distribution of items within each factor. This underscores the importance of investigating the reliability and validity of the EAT-26 in the targeted population. Nonetheless, explanations for the varying factorial structure of the EAT-26 have been offered in the literature including the type of sample (e.g., clinical vs non-clinical) [56], cultural differences (e.g., ideal body image and different standards for beauty, norms and values, social pressure regarding eating habits).

Despite an extensive literature search, no publication was found that investigated the factorial structure of the EAT-26 specifically among Saudi samples. This is concerning since recent reviews have revealed that the EAT-26 is the most commonly utilised tool in research screening for EDs and DEBAs in this under-represented and under-researched population [5, 7, 45] and given the high prevalence of obesity and DEBAs among the Saudi people [6, 8, 24, 28]. Reliable and valid screening tools becomes of high importance for research and clinical use. Consequently, this study aimed to resolve inconsistencies in the factorial structure of the Arabic version of the EAT-26 by identifying the best-fitting model and evaluating its measurement invariance across sexes and Body Mass Index (BMI) categories in a large non-clinical Saudi sample.

## Methods

### Participants

This study formed a crucial part of a larger research project that delved into the intricate relationships between clinical perfectionism, depression, anxiety, and disordered eating behaviours among adults from the general population in Saudi Arabia (for example see [11]). A total of 1,734 Saudi participants completed an online anonymous survey. To take part in this research, participants had to be Saudi adults aged 18 years or older and native Arabic speakers. The age range was from 18 to 77 years (*M*_age_ 26.88 and SD 9.13), with 78.4% of participants being female. Participants were from all 13 main regions in Saudi Arabia, with the majority from Mecca region (42.9%) followed by Riyadh region (24.3%), Eastern region (10.6%), … and Al-Jowf region (0.6%). The average BMI was 24.78 (SD 6.25). Among the respondents, 48.6% had a normal weight, 17.2% were classified as obese, 22.7% were overweight, and 11.5% were underweight.

### Procedure

A cross-sectional online study was conducted between October 2020 and January 2021. Participants were recruited through a social media post that included a link to the study on Google Forms, shared across the research team's accounts on platforms such as X, Facebook, WhatsApp, and Telegram student groups, employing convenience and snowball sampling techniques. All survey questions were made mandatory, meaning there could be no missing data, and participants were only able to submit their responses after completing all questions. In line with standard practices, participation was voluntary, and all participants provided electronic informed consent. No identifying information was collected and access to the dataset was restricted to the research team. No incentives were offered to the participants. The study received ethical approval from the university ethics review board.

### Measures

Participants provided demographic information including age, sex, and area of residence, and self-reported their weight (kg) and height (cm), which were used to calculate BMI according to the Center for Disease Control and Prevention guidelines (BMI < 18.5 underweight; BMI ≥ 18.5 and < 25 healthy weight; BMI ≥ 25 and < 30 overweight; and BMI ≥ 30 obesity) [18].

Depressive and anxiety symptoms were assessed using the Arabic versions of the Patient Health Questionnaire (PHQ-9) and the General Anxiety Disorder-7 (GAD-7) respectively [38, 57]. Both scales were scored on 4-point Likert scale ranging from 0 (not at all) to 3 (nearly every day) Scores for individual items on each measure were added to obtain a composite score, with a possible range of 0 to 27 for the PHQ-9 and 0 to 21 for the GAD-7. Higher scores on the PHQ-9 and GAD-7 indicate increased symptoms. The Arabic version of the PHQ-9 [4] and GAD-7 [3] have demonstrated robust psychometric properties in Saudi samples. In the current study, the Cronbach's alpha (α) was 0.88 for PHQ-9 and 0.91 for GAD-7. Participants also completed the Arabic version of the EAT-26, which is described in more detail below.

### Eating attitudes test

The EAT-26 comprise three subscales according to the original English version: (1) dieting (13 items), (2) bulimia and food preoccupation (6 items), and (3) oral control (7 items) [27]. The first 25 items are scored using a 4-point Likert scale with the following scoring options: never, rarely, and sometimes (0), often (1), usually (2), and always (3). The final item (item number 26) is reversed-scored. A total score is calculated by summing all items’ scores (range from 0 to 78). A score ≥ 20 indicates possible disordered food attitudes [27]. The Arabic version used in this study was adapted from [36], who translated the EAT-26 into Arabic and evaluated its factor structure using exploratory factor analysis (EFA), ESEM, and confirmatory factor analysis (CFA) in a large sample of young Qatari female university students. Khaled et al. [36] found that a 19-item five-factor model demonstrated the best fit with internal consistency ranging from 0.72 to 0.84.

### Data analysis

The data were analysed using the SPSS 26.0 [32]) and Amos 26.0 software programs [12]. Prior to conducting CFA, the assumption of normality was checked. Both univariate and multivariate normality were examined by obtaining skewness and kurtosis values, as well as Mardia’s normalized multivariate kurtosis coefficient. Skewness and kurtosis values for all items, except for one (item 9: skewness = 3.88, kurtosis = 14.27) were within the expected range for normality. Evidence of multivariate non-normality was found, with Mardia’s coefficient for multivariate kurtosis for all items of the EAT was 183.61. Consequently, successive CFAs the Bollen-Stine bootstrap strategy (2000 samples), along with a bi-as-corrected confidence interval (90% CI) and a maximum likelihood estimation method was employed to assess the factor structure of the EAT-26 Arabic version. Several existing models were tested including the original 26-item three-factor model [27], second order 26-item three-factor model, 20-item four-factor model [37], and 16-item four-factor model [51]. The model fit was evaluated using several goodness-of-fit indices: the comparative fit index (CFI), standardized root mean square residual (SRMR), and root mean square error of approximation (RMSEA). The model was considered a good fit if the CFI was greater than or equal to 0.90, and the SRMR and RMSEA were less than or equal to 0.08 [15, 16, 31]. Descriptive statistics of the EAT were calculated and internal consistency was assessed using Cronbach’s α and McDonald’s omega (ω) coefficients [21, 42]. Cronbach’s α is widely recognized and easy to calculate, while ω coefficient offers greater accuracy for complex factor structures. Both coefficients are interpreted in a similar manner, where a coefficient of 0.7 or more indicates acceptable reliability.

Next, a multi-group confirmatory factor analysis (MGCFA), probably the most widely used approach to test measurement invariance between groups [35], was used to test the measurement invariance across sexes and BMI categories. Three levels of invariance were tested including configural, metric, and scalar invariance [20]. Configural invariance indicates that the factor structure is the same between the comparison groups. Metric invariance implies that the factor loadings for similar items are equivalent across groups. Scalar invariance means that the item intercepts are equivalent across groups [35]. Evidence for invariance was determined if the changes in CFI (ΔCFI) were less than or equal to 0.01, the changes in RMSEA (ΔRMSEA) were less than or equal to 0.015, and the changes in SRMR (ΔSRMR) were less than or equal to 0.03 for tests of metric invariance. For tests of scalar invariance, the criteria were ΔCFI ≤ 0.01, ΔRMSEA ≤ 0.015, and ΔSRMR ≤ 0.01 [17, 19].

## Results

### Factorial validity of EAT

CFA was conducted using the maximum likelihood estimation method to assess the original three-factor structure of EAT-26 [27]. CFA results indicated that the model fit was unacceptable for the original three-factor model (CFI = 0.727; SRMR = 0.0911; RMSEA = 0.085 [90% CI 0.082–0.087]).

Three alternative models from the literature were also tested. Second order 26-item three-factor model, Koslowsky et al.’s [37] 20-item four-factor model, and Ocker et al.’s [51] 16-item four-factor model. The results of the individual model fit indices are shown in Table 1. The results indicated that both second order three-factor and Koslowsky et al.’s [37] models provided a relatively poor fit. Ocker et al.’s [51] 16-item four-factor model demonstrated an acceptable fit (CFI = 0.904, SRMR = 0.0554; RMSEA = 0.073 [90% CI 0.068- 0.–77]). The standardized factor loadings are shown in Table 2. All factor loadings were significant at *p* < 0.001 and ranged from 0.44 to 0.83. As a result, the psychometric properties of the 16-item four-factor model were assessed in all subsequent analyses. The revised Arabic EAT-16 version is in the Online Appendix.

**Table 1:** Fit indices of the original and alternative models of the Arabic EAT-26

Model	*χ*^*2*^ (*df*)	*p*	CFI	RMSEA (90% CI)	SRMR
Original three-factor model	3959.44 (296)	0.000	0.727	0.085 (.082–.087)	0.0911
Second order three-factor model	3896.12 (297)	0.000	0.732	0.084 (.081–.086)	0.0913
20-item four-factor model	1786.20 (164)	0.000	0.847	0.076 (.072–.079)	0.0800
16-item four-factor model	992.16 (98)	0.000	0.904	0.073 (.068–.077)	0.0554

*df* = degrees of freedom; CFI = comparative fit index; RMSEA = root mean square error of approximation; CI = confidence interval; SRMR = standardized root mean square residual

**Table 2:** Descriptive statistics, reliability coefficients, and standardized factor loading for the Arabic EAT-16 version

Factor	Item (number on the EAT-26)	Mean (SD)	Factor loading	α	ω
Self-perception of Body Shape	I am terrified about being overweight (1)	1.13 (1.22)	0.66	0.66	0.71
	I am occupied with a desire to be thinner (11)	1.16 (1.30)	0.83		
	I am preoccupied with the thought of having fat on my body (14)	0.70 (1.12)	0.44		
Dieting	I feel extremely guilty after eating (10)	0.63 (1.07)	0.66	0.74	0.74
	I think about burning up calories when I exercise (12)	1.16 (1.29)	0.69		
	I feel uncomfortable after eating sweets (22)	0.77 (1.14)	0.56		
	I engage in dieting behavior (23)	0.75 (1.10)	0.58		
	I Like my stomach to be empty (24)	0.67 (1.05)	0.48		
Awareness of Food Contents	I am aware of the calorie content of foods that I eat (6)	0.44 (0.91)	0.61	0.75	0.75
	I particularly avoid food with a high carbohydrate content (i.e. bread, rice, potatoes, etc.) (7)	0.38 (0.85)	0.66		
	I avoid foods with sugar in them (16)	0.52 (0.94)	0.62		
	I eat diet foods (17)	0.43 (0.88)	0.73		
Food Preoccupation	I find myself preoccupied with food (3)	0.65 (1.04)	0.69	0.80	0.80
	I have gone on eating binges where I feel that I may not be able to stop (4)	0.60 (1.02)	0.69		
	I feel that food controls my life (18)	0.60 (1.05)	0.78		
	I give too much time and thought to food (21)	0.59 (1.03)	0.67		
EAT-16 total Score		11.20 (10.10)	–	0.88	0.88

SD, standard deviation; α, Cronbach alpha, ω, McDonald omega. All factor loadings were significant at *p* < .001

### Descriptive analysis, reliability, and correlations

Descriptive statistics and reliabilities coefficients of the EAT-16 are indicated in Table 2. The mean score of the EAT-16 was 11.20 (SD 10.10). The inter-factor correlations for the four identified factors (Self-Perceptions of Body Weight, Dieting, Awareness of Food Contents, and Food Preoccupation) were between 0.28 and 0.68. The revised Arabic EAT-16 showed good internal consistency (Cronbach’s α and McDonald’s ω were 0.88). Reliability coefficients for the individual factors are presented in Table 2. Furthermore, the EAT-16 demonstrated convergent validity through significant positive correlations with measures of depression (*r* = 0.20, *p* < 0.001) and anxiety (*r* = 0.22, *p* < 0.001). These associations were within the expected magnitude and direction.

### Measurement invariance across gender and weight status

Results MGCFA analyses across sexes and four BMI categories (underweight, normal weight, overweight, and obese) are shown in Table 3. The configural model to the data was acceptable between males and females (CFI = 0.903, SRMR = 0.0557, RMSEA = 0.052 [90% CI = 0.049–0.055]). The metric (ΔCFI = 0.001, ΔRMSEA = 0.002, ΔSRMR = 0.0000) and scalar (ΔCFI = 0.000, ΔRMSEA = 0.001, ΔSRMR = 0.0001) invariance models were also supported, suggesting measurement invariance between males and females.

**Table 3:** Measurement invariance of the EAT-16 across sexes and BMI categories

	Overall fit indices					Comparative fit indices				
Model	*χ*^*2*^ (*df*)	*p*	CFI	RMSEA (90% CI)	SRMR	Δ*χ*^*2*^ (*df*)	*p*	ΔCFI	ΔRMSEA	ΔSRMR
Sex^1^										
Configural	1102.68 (196)	0.000	0.903	0.052 (.049-.055)	0.0557					
Metric	1125.11 (208)	0.000	0.902	0.050 (.048-.053)	0.0557	22.43 (12)	0.032	0.001	0.002	0.0000
Scalar	1137.25 (220)	0.000	0.902	0.049 (.046-.052)	0.0556	12.15 (12)	0.433	0.000	0.001	0.0001
BMI categories^2^										
Configural	1315.80 (392)	0.000	0.902	0.037 (.035-.039)	0.0752					
Metric	1353.06 (428)	0.000	0.902	0.035 (.033-.037)	0.0751	37.26 (36)	0.411	0.000	0.002	0.0002
Scalar	1374.86 (464)	0.000	0.903	0.034 (.032-.036)	0.0750	21.8 (36)	0.970	0.001	0.001	0.0001

*df* = degrees of freedom; CFI = comparative fit index; RMSEA = root mean square error of approximation; CI = confidence interval; SRMR = standardized root mean square residual; ^1^ Male vs. Female; ^2^ Underweight, Normal weight, Overweight, and Obese

With regards to the BMI categories, the configural model was supported (CFI = 0.902, SRMR = 0.0752, RMSEA = 0.037 [90% CI = 0.035–0.039]). The metric (ΔCFI = 0.000, ΔRMSEA = 0.002, ΔSRMR = 0.0002) and the scalar (ΔCFI = 0.001, ΔRMSEA = 0.001, ΔSRMR = 0.0001) invariance models were also supported. These results suggest that the factor loadings were equivalent across the different BMI categories and that responders, across the different BMI categories, interpreted and responded to the items in the same way. Taken together, all levels of measurement invariance were achieved across sexes and BMI categories.

## Discussion

This research explored the factorial structure, reliability, and measurement invariance across sexes and BMI categories of the Arabic version of the EAT-26 in a large non-clinical Saudi sample. By systematically testing existing factorial models of the EAT-26, this study adds to the literature by identifying the most robust and empirically supported structure among the Saudi general adult population. Successive CFAs revealed poor fit indices for the original three-factor model [27], second order 26-item three-factor model, Koslowsky et al.’s [37] 20-item four-factor model. Instead, CFA identified an optimal 16-item, four-factor structure (Self-Perceptions of Body Weight, Dieting, Awareness of Food Contents, and Food Preoccupation) of the Saudi Arabic version of the EAT-26. This revised 16-item Arabic version had good internal consistency as assessed by Cronbach’s α and McDonald’s ω coefficients. Furthermore, measurement invariance analyses of the 16-item version supported configural, metric, and scalar invariance across sexes and BMI categories.

The revised EAT-16 Saudi Arabic version is consistent with Ocker’s et al. (2007) model, in terms of the number of factors and number of items. In their study, Ocker’s et al. (2007) evaluated the model fit of the 26-item original three-factor model [27] and a 20-item, four-factor model [37] using two independent female college samples (a calibration sample and a cross-validation sample). Their analyses showed that even though the Koslowsky’s et al. [37] model had a relatively better fit compared to the original three-factor model [27], the RMSEA and CFI values did not meet the acceptable standards. After removing the four problematic items with low factor loadings, a four-factor model with 16 items was found to have an adequate fit [51]. Our findings advance our understanding of the psychometric properties of EAT-26 in a non-Western context through identifying the best-fitting model for the Saudi general adult population and thus providing a unique contribution to the EAT-26 literature.

Measurement invariance, often neglected in applied research [40], is important as it sheds light on whether group differences in a given psychological construct (a latent variable such as DEBAs) can be attributed to real differences in the underlying construct being measured, rather than being due to biases or problems with the measurement instrument itself [35]. The EAT-16 Arabic version demonstrated configural, metric, and scalar invariance indicating that the factor structure, factor loadings, and item intercepts are equivalent across sexes and the BMI categories (underweight, normal weight, overweight, and obese), and therefore measuring DEBAs in the same way across these groups. This finding is consistent with previous research providing evidence for measurement invariance for an 18-item EAT French version across gender, ethnicity, age and BMI groups [41], a 21-item EAT Spanish version across gender [48], and for the same model tested in the current research (a 16-item, four-factor model English version) across Caucasian and Hispanic ethnicities [13]. Overall, researchers using the Arabic EAT-16 in the Saudi general population can be assured that this measure is psychometrically equivalent across sexes and BMI categories.

The invariance of EAT-16 scores across sexes and BMI categories has implications for both research and clinical practice. This consistency enhances the generalizability of findings in studies utilizing EAT-16 among Saudi adult samples, allowing for meaningful comparisons. Clinically, the EAT-16 serves as a screening tool for DEBAs, enabling targeted interventions that focus on changing DEBAs rather than demographic factors. This promotes a holistic approach to identification and treatment, ensuring that care is relevant and accessible to diverse populations.

The current study found small but significant positive correlations between total scores on the EAT-16, PHQ-9 (a measure of depression) and GAD-7 (a measure of anxiety), providing evidence for convergent validity. This finding is consistent with previous studies by Haddad et al. [30] and McLean et al. [44], which also reported positive correlations between measures of DEBAs, depression, and anxiety.

Overall, given the widespread use of the EAT-26 to assess DEBAs among Arabic-speaking populations, especially Saudi samples [5, 7, 45], the current study has important implication. The original three-factors EAT-26 was developed in a Western context [27], which may not fully capture the nuances of DEBAs in non-Western contexts. The revised and culturally adapted Arabic EAT-16 would improve screening for and identifying individuals at risk of EDs within the Saudi general population. This is crucial for enhancing early detection and facilitating timely intervention.

### Limitations and future research

The present study has several strengths. First, it examined the psychometric properties of the Arabic version of the EAT-26 in Arabic-speaking general sample, and thus, provided empirical evidence for a revised version of the EAT-26 that is culturally relevant and empirically supported for use in this population. Second, previous research has primarily focused on female adolescents and adults [13, 34, 36, 47], precluding measurement invariance testing across genders. In contrast, the present study included participants of both sexes and successfully established measurement invariance. Thus, providing evidence that the four-factor model holds across sexes as well as BMI categories (underweight, normal weight and overweight and obese).

However, a few limitations should be noted. First, our sample consisted of Saudi adults from the general population and therefore our findings may not generalize beyond this population (e.g., young adults and individuals with mental health disorders including EDs). Second, height and weight were self-reported for BMI, which are subject to bias and inaccuracies. Future research should use objective measurements of height and weight. Future research should also test whether this revised 16-item EAT version holds in clinical samples and samples with age heterogeneity. The present study also did not assess other important psychometric properties including temporal stability, other forms of validity (e.g., criterion and discriminant validity). Future research should consider investigating these psychometric parameters to gain more insight into the measure's reliability and validity. Future work could also consider using item response theory models such as the Rasch analysis to evaluate the performance and unique contribution of individual items and identify those that do not fit the underlying latent construct well.

## Conclusion

In summary, the present study provided evidence for a revised 16-item, four-factor model of the EAT-26 to measure DEBAs among Saudi general adult population. This revised model demonstrated good internal consistency, convergent validity, and measurement invariance. Researchers and clinicians should avoid using the original EAT-26 and instead use this newly proposed 16-item version in this demographic. Future research should examine the factorial structure and other psychometric parameters such as criterion and discriminant validity of this revised model in clinical settings to further establish its utility for the identification and evaluation of eating-related concerns within Arabic cultural contexts.

## Supplementary Information

Additional file1 (DOCX 23 KB)

## Data Availability

Data associated with this manuscript can be retrieved from 10.17605/OSF.IO/8ET5Q.

## Abbreviations

EAT-26: The eating attitude test 26-item version
GAD-7: The general anxiety disorder scale
PHQ-9: The patient health questionnaire
BMI: Body mass index
DEBAs: Disordered eating behaviours and attitudes
EDs: Eating disorders
EFA: Exploratory factor analysis
CFA: Confirmatory factor analysis
ESEM: Exploratory structural equation modelling
MGCFA: Multi-group confirmatory factor analysis
SNMHS: Saudi National mental health survey
