# Supplementary material for: Assessing disordered eating behaviours and attitudes: Factor structure and measurement invariance of the Arabic version of the eating attitudes test (EAT-26) in Saudi Arabia
Source: J Eat Disord. 2024 Nov 19;12:185. doi: 10.1186/s40337-024-01137-2 (PMC11577874; doi:10.1186/s40337-024-01137-2)
Supplement: Supplementary file 1 — Additional file1 (DOCX 23 KB) [file 40337_2024_1137_MOESM1_ESM.docx]

**Appendix**

**Arabic EAT-16 with their corresponding English items**

|  | **Item** | **Item number on EAT-26** | **عبارة** |  |
| --- | --- | --- | --- | --- |
| 1 | I am terrified about being overweight | 1 | أنا أرتعب من أن يكون وزني زائدا | 1 |
| 2 | I am occupied with a desire to be thinner | 11 | أنا منشغل/منشغلة برغبتي في أن أكون نحيف/نحيفة | 2 |
| 3 | I am preoccupied with the thought of having fat on my body. | 14 | أنا منهمك/منهمكة في التفكير في تكدس السمنة بجسمي | 3 |
| 4 | I feel extremely guilty after eating | 10 | أشعر بذنب شديد بعد الاكل | 4 |
| 5 | I think about burning up calories when I exercise | 12 | أفكر في حرق السعرات الحرارية حين أمارس الرياضة البدنية | 5 |
| 6 | I feel uncomfortable after eating sweets | 22 | أشعر بعدم الارتياح بعد تناول الحلويات | 6 |
| 7 | I engage in dieting behavior | 23 | أتبع حمية غذائية معينة | 7 |
| 8 | I like my stomach to be empty | 24 | أحب أن تكون معدتي فارغة | 8 |
| 9 | I am aware of the calorie content of foods that I eat | 6 | أنا على علم بما تحتويه الأطعمة التي آكلها من سعرات حرارية | 9 |
| 10 | I particularly avoid food with a high carbohydrate content (i.e. bread, rice, potatoes, etc.) | 7 | أتجنب الأطعمة الغنية بالنشويات خصوصاً (مثل الخبز والأرز والبطاطا...) | 10 |
| 11 | I avoid foods with sugar in them | 16 | أتجنب الأطعمة التي تحتوي على السكر | 11 |
| 12 | I eat diet foods | 17 | أتناول الأطعمة الخاصة بالحمية الغذائية | 12 |
| 13 | I find myself preoccupied with food | 3 | أجد نفسي منشغل/منشغلة بالطعام | 13 |
| 14 | I have gone on eating binges where I feel that I may not be able to stop | 4 | سبق أن مررت بنوبات من الإفراط في الأكل حيث أشعر أننى عاجز/عاجزة عن التوقف | 14 |
| 15 | I feel that food controls my life | 18 | أشعر بأن الطعام يتحكم بحياتي وبسلوكي | 15 |
| 16 | I give too much time and thought to food | 21 | أكرس نسبة كبيرة من الوقت والتفكير للطعام | 16 |
